# Alpha-1 Antitrypsin Deficiency and Pulmonary Morbidity in Patients with Primary Immunodeficiency Disease: A Single-Center Experience

**DOI:** 10.1155/2020/4019608

**Published:** 2020-05-27

**Authors:** Georg Evers, Arik Bernard Schulze, Michael Thrull, Jan-Philipp Hering, Christoph Schülke, Rainer Wiewrodt, Helmut Wittkowski, Lars Henning Schmidt, Michael Mohr

**Affiliations:** ^1^Department of Medicine A, Hematology, Oncology and Pneumology, University Hospital Muenster, Muenster, Germany; ^2^Department of Clinical Radiology, University Hospital Muenster, Muenster, Germany; ^3^Department of Pediatric Rheumatology and Immunology, University Hospital Muenster, Muenster, Germany

## Abstract

**Background:**

Alpha-1 antitrypsin deficiency (AATD) is of importance in the pathogenesis of pulmonary emphysema, chronic obstructive pulmonary diseases (COPD), and bronchiectasis. Various pulmonary disorders are a typical feature of primary immunodeficiency disease (PID). This includes recurrent pulmonary infections, immunodysregulation, and autoinflammatory diseases. As a result, incidence of acute and chronic pulmonary diseases is higher. Interestingly, pulmonary morbidity in PID and AATD share similar features. To study the coexistence of AATD in patients suffering from PID, we performed the underlying investigation.

**Methods:**

We evaluated a study group of 149 patients (*n* = 149) with PID. In total, serum AAT concentrations were available for 110 patients (*n* = 110). For the identified patients, we analyzed both clinical associations and interactions.

**Results:**

Among the investigated patients, reduced serum AAT levels were detected in 7 patients. With regard to the genotype, PI∗ZZ was found in 2 patients, whereas PI∗MZ was observed in 5 patients. Independent of the underlying phenotype, obstructive lung diseases were found in 2 patients with PI∗ZZ and 2 patients with PI∗MZ.

**Conclusions:**

In Germany, the estimated percentage for PI∗ZZ and PI∗MZ is 0.01% and 1.9%, respectively. As demonstrated, the ratio in our study group was even higher. We identified seven patients with AATD. Since AATD contributes to pulmonary morbidity in PID patients, systematic underdiagnosis of the coexistence might yield a strong clinical impact. Hence, AAT analysis should be offered to all patients with confirmed PID diagnoses. To strengthen this finding, we suggest the investigation of larger databases.

## 1. Introduction

The term “primary immunodeficiency disease” (PID) encompasses a heterogeneous group of diseases with underlying and often inherited immune defects. So far, various isolated defects and combined disorders are differentiated, including humoral immunodeficiencies, severe combined immunodeficiencies, and disorders from phagocytic and complement defects [1]. In adults, the group of primary antibody deficiencies represents more than 80% of PID cases. According to the current European Society for Immunodeficiency (ESID) criteria, besides other humoral immunodeficiencies such as unclassified antibody deficiency, selective IgA/IgM deficiency, and isolated IgG subclass deficiency, common variable immunodeficiency disorder (CVID) is the most common symptomatic antibody deficiency, affecting approximately 20% of PID patients [2, 3]. Due to antibody deficiency, patients often suffer from recurrent upper and lower respiratory tract infections [4].

The spectrum of pulmonary morbidity comprises both obstructive and restrictive lung disease as well as bronchiectasis due to frequent infections [5]. Recurrent respiratory infections mostly represent the first manifestation of PID and should lead to further immunologic evaluation. The assessment of suspected primary humoral immunodeficiency includes quantitative immunoglobulin levels, specific antibody titers, and the enumeration of B-cells and B-cell subpopulations.

Alpha 1-antitrypsin deficiency (AATD) is an inherited and clinically underrecognized disorder [6]. Since AAT inactivates proteolytic enzymes in pulmonary tissues, AATD results in an imbalance between proteases and antiproteases which favors tissue destruction and thus might yield pulmonary morbidity such as chronic obstructive diseases, emphysema, and bronchiectasis [7, 8]. Depending on AATD serum levels, tobacco smoking, or occupational air pollutants can even accelerate the development of clinical manifestations dramatically [9]. To treat this issue, patients with evidence of airflow limitation or emphysema from AATD might be considered for AAT augmentation. These patients often have an AAT serum level below 11 micromol/L (approximately 50 mg/dl) with an underlying AAT genotype PI∗ZZ [10, 11]. Moreover, due to abnormalities in tertiary structure of the molecule, AAT can accumulate in hepatocytes leading to cell injury followed by fibrosis and cirrhosis [12].

Diagnosis of AAT deficiency (AATD) is confirmed if both low AAT levels and specific defective genotypes are found [13]. Genetically, AATD is an autosomal recessive disease. The SERPINA1 gene encodes AAT and is located on the long arm of chromosome 14. Due to its pleomorphic character, at least 150 alleles of the AAT gene have been identified. In general, the allelic variants are classified according to their effects on AAT serum concentration. Normal alleles (M1 to M6) are associated with normal protein functions. Deficient alleles are accompanied by decreased serum AAT concentrations.

The majority of patients who suffer from lung or liver diseases are homozygous for the alleles Z or S (PI∗ZZ and PI∗SS genotype). Interestingly, genotype PI∗SS is not associated with an increased risk for developing a lung disease, whereas PI∗SZ heterozygotes are more likely to develop a lung disease, especially if they smoke [14]. Patients with genotype PI∗00 are a rarity but develop the most severe forms of pulmonary morbidity without developing liver disease. In contrast, in other heterozygous forms of AATD (PI∗MS and PI∗MZ), serum AAT levels are less decreased, and data are conflicting regarding the risk of pulmonary morbidity, with some studies suggesting increased risk for pulmonary morbidity in smokers with MZ phenotype [15, 16].

Globally, Caucasians in North America and Europe are considered to have the highest allele frequencies for AATD. In Germany, for instance, estimated percentage for AAT genotype PI∗ZZ and PI∗MZ is 0.01% and 1.9%, respectively [17]. However, the anticipated prevalence is thought to be higher than the number of patients with clinically identified AATD.

The pathophysiology of AATD is not clarified yet. For instance, almost 35% of all patients with PI∗ZZ genotype do not show any clinical symptoms at all [18]. Independent of protease inhibition, AAT helps in immune regulation and influences inflammatory processes in the lung. Moreover, AATD has been reported in patients with hypogammaglobulinemia or other PID (Table 1).

Following the identification of one patient with both PID and concomitant AATD, we investigated a larger study group of PID patients with focus on the coexistence of PID and AATD.

## 2. Material and Methods

### 2.1. Systematic Review of the Literature

**Figure 1:**
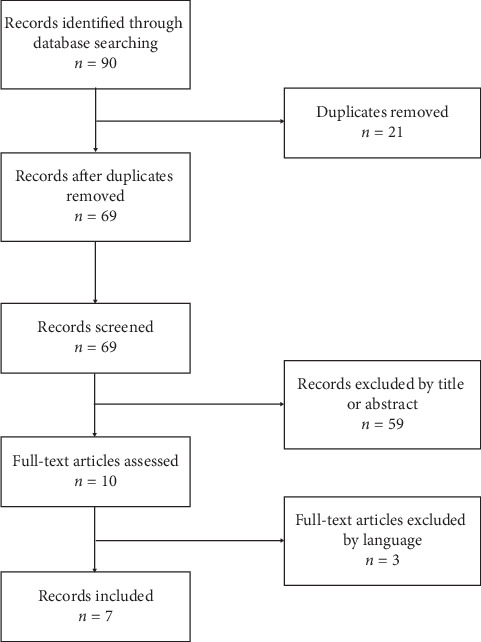
PRSIMA diagram of the literature search.

Systematic literature search of various articles was performed by two authors independently of one another. MEDLINE and Cochrane Library databases were checked for the following keywords: “alpha 1-antitrypsin deficiency,” “hypogammaglobulinemia,” “primary immune deficiency,” “primary immunodeficiency,” “CVID,” “primary humoral immunodeficiency,” “unclassified antibody deficiency,” “selective IgM deficiency,” “selective IgA deficiency,” “isolated IgG subclass deficiency,” and “IgG subclass deficiency with IgA deficiency.” There were not any time limits set in the literature search, but only articles written in English were taken into account. Duplicates were censored. Publications about diagnosis of AATD coexistent with primary immune deficiency, together with patients of the current study, are summarized in Table 1. The review process is summarized in Figure 1.

### 2.2. Study Population

Before data collection, approval from the joint Ethical Committee of the Faculty of Medicine of the Westphalian Wilhelms-University Muenster and the Physicians Chamber of Westphalia-Lippe was obtained (application number: 2015-376-f-S). Since data collection was retrospective, written consent was not necessary. All included patients were medically treated in the Department of Medicine A, Pulmonary Division, University Hospital Muenster, Germany, between 2010 and 2017. Our Pulmonary division offers a special consultation for patients with PID or suspected PID. Diagnostics in patients with suspected immunodeficiency are based on the interdisciplinary guideline for the diagnostics of primary immunodeficiency of the Association of the Scientific Medical Societies (AWMF) in Germany [26]. In total, 149 PID patients, meeting international (ESID) diagnostic criteria, were identified.

**Figure 2:**
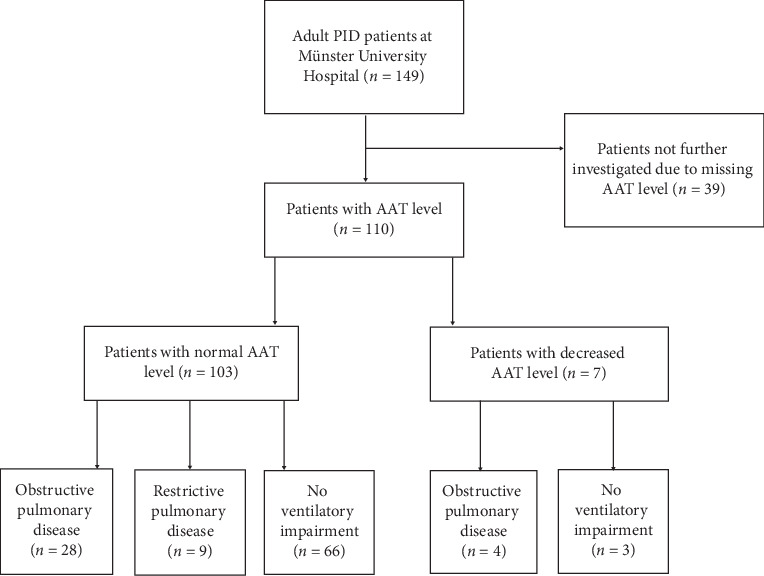
Study group and clinical subgroups.

Serum alpha-1 antitrypsin levels were measured within the routine laboratory in 110 of 149 patients (74%), using nephelometry on Beckmann Array™ Protein system. For all patients, information such as lung function and laboratory and radiological parameters was gathered. Apart from one patient with reduced AAT level, patients with AATD established by serum AAT levels below a threshold value of ≤90 mg/dl were further investigated by genotyping and phenotyping (Figure 2). This threshold was considered as the lower limit of normal in our laboratory at University Hospital Muenster. In a recent international guideline, a threshold of 24.4 micromol/L (110 mg/dl) was considered to be optimal to discriminate normal PI∗MM from genotypes carrying at least one deficient S or Z allele. We have therefore checked to see if we should have tested more patients at a higher threshold of 110 mg/dl, but this was not the case.

Genotyping of the protease inhibitor locus was performed on a dried blood sample (DBS, Alpha Kit) at the AAT laboratory at University Hospital Marburg.

### 2.3. Radiological Imaging

Pulmonary morbidity (i.e., bronchiectasis, emphysema) was assessed in PID patients by expert radiologists in the Department of Radiology, University Hospital Muenster, using CT-scanning or chest X-ray if indicated clinically due to recurrent chest infections. PID patients without history of recurrent pulmonary infections or any ventilatory limitation using spirometry were not further investigated by radiological imaging. With respect to PID patients with AATD, in five out of seven individuals, thoracic computed tomography or chest X-ray was performed to evaluate lung morphology.

### 2.4. Statistical Analysis

The study population was described by standard descriptive statistical measures. Absolute and relative frequencies are reported for categorical variables. For continuous variables, median and interquartile ranges are reported, respectively. Association of clinicopathological parameters with AATD patients was investigated. Therefore, *P* values for categorical variables were calculated using either Chi-square test or Fisher's exact test.

All statistical tests were performed as exploratory analyses on a local significance level of 0.05. Since multiplicity adjustment was not carried out, no distinct overall significance level was ascertained. Hence, our findings may be used to set up new hypotheses. The statistical software (SPSS Statistics, Version 25.0, released 2017, IBM Corp., Armonk, NY) was used for all analyses.

## 3. Results

### 3.1. Study Population

The entire study population contains 149 patients (*n* = 149), who attended our clinic with PID between 2010 and 2017. In total, 110 PID patients (*n* = 110) had a serum AAT determination and were further evaluated.  Table 2 summarizes baseline characteristics of the investigated study population. Clinical data was gathered such as age, exact PID diagnosis (according to ESID criteria), immunoglobulin and AAT level, and radiologic parameter (e.g., bronchiectasis). With regard to functional impairment (i.e., obstructive or restrictive lung disease) or bronchiectasis, 38 patients (35%) had clinical symptoms, 32 patients (29%) suffered from obstructive airway diseases, and restrictive lung diseases were found in 8 patients (8%). The number of patients with bronchiectasis was higher in the group with obstructive pulmonary diseases.

In 110 PID patients with measured AAT levels, prevalence of individuals with PI∗ZZ phenotype and resultant severe deficiency of AAT was 1.8% (*n* = 2). Mildly reduced AAT serum levels were observed in 5 patients (4.5%). Further evaluation revealed PI∗MZ heterozygosity in each of these patients. Both patients with homozygous AATD suffered either from unclassified antibody deficiency or from CVID. Heterozygous variants of AATD were found to be coexistent in two patients with selective IgM deficiency and in one patient with CVID, unclassified antibody deficiency, and isolated IgG subclass deficiency.

With focus on clinicopathological correlations between the three subgroups of PID patients with 2 patients with PI∗ZZ, 5 patients with PI∗MZ, and 103 patients without AATD, the number of cases was too small to determine statistical differences between subgroups.

Assessment of pulmonary morbidity by expert radiologists did not identify common pathologic patterns in patients with either homozygous or heterozygous AAT.

### 3.2. Presentation of Two Cases of Patients with Coincident AATD and PID

Patient #1 is a 25-year-old male Caucasian with a diagnosis of AATD PI∗ZZ and AAT serum level of 19.50 mg/dl (90–200 mg/dl). Owing to persistent hypogammaglobulinemia with recurrent infections (IgG: 680 mg/dl (700–1600); IgA 47 mg/dl (70–400); IgM: 22 mg/dl (40–230)) and poor response to pneumococcal polysaccharide-based vaccine, CVID was diagnosed. Although the number of CD4+ T-cells was slightly reduced, the ESID criteria for CVID were met. Further testing for genetic defects in CVID was carried out, but clinical relevance remains unclear so far. Besides a history of atopic dermatitis, various allergies were known. Due to recurrent upper and lower respiratory tract infections, immunoglobulin substitution was recommended but, nevertheless, this has been rejected by the patient so far. However, lung function parameters remained stable without evidence of obstructive or restrictive disease.

**Figure 3:**
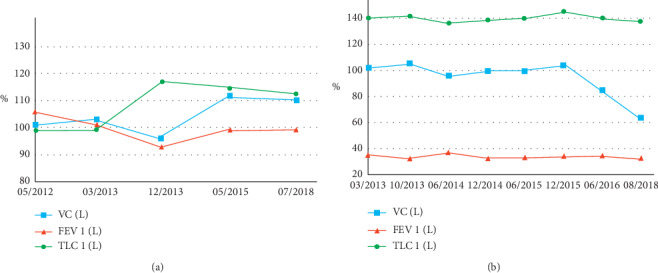
Lung function in the course of disease of patient #1 (a) and patient #2 (b).

Patient #2 is a 44-year-old male Caucasian ex-smoker (15 pack years), who was referred to our outpatient department with already known diagnosis of AATD PI∗ZZ and recurrent infections of the upper and lower respiratory tract. Because of severe obstruction (FEV1: 1.46l, 32%), AAT augmentation therapy was already started with an AAT serum level of 57 mg/dl under substitution. Due to a history of recurrent infections and suspected humoral immune deficiency, quantitative measurement of total serum immunoglobulin was performed showing markedly reduced IgG-concentration (IgG: 571 mg/dl (700–1600); IgA 205 mg/dl (70–400); IgM: 75 mg/dl (40–230)) consistent with diagnosis of unclassified antibody deficiency. Moreover, immunophenotyping revealed severe deficiency for memory B-cells. Immunoglobulin replacement therapy markedly reduced infection frequency, and further deterioration of lung function parameters was prevented (Figure 3).

Notably, on the basis of exonic and splice-site variants, next generation sequencing was performed in both patients, but no common genetic features could be identified. In patient #1, the following heterozygous variants with an allele frequency of <1% were found: VAV2, TCF3, CLEC16A, and LRBA. In patient #2, heterozygous variants with an allele frequency of <1% were identified for TNFRSF16, AKT1, TNFRSF17, NLRP12, and TCF3. Variants of CLEC16A are associated with selective IgA deficiency and CVID [27]. However, clinical relevance of these findings remains unclear in the context of CVID/PID and AATD so far.

## 4. Discussion

In the underlying study we investigated a study group of 110 patients diagnosed with several types of PID disorders with focus on serum AAT levels and other clinical parameters. Among the investigated patients, reduced serum AAT levels (≤90 mg/dl) were found in 7 patients. With focus on phenotype, further examination revealed phenotype ZZ in two patients, and phenotype MZ was found in 5 patients.

### 4.1. Prevalence of AATD and PID

In previous publications, a possible association between AAT deficiency and hypogammaglobulinemia was postulated, too. The idea originally derived from mere theoretical assumptions including reports of patients with concomitant AATD and hypogammaglobulinemia [22, 23], coexistence of bronchiectasis/emphysema in patients with either AATD or CVID [28], and the local proximity of the genes for the immunoglobulin heavy chain and AAT [29].

In contrast, elevated free light chains (FCLs) have been associated with chronic inflammation, such as COPD. In addition, a biomarker role in AATD has also been considered [30]. In this context, increased free light chains not only have been observed in chronic inflammation, but may also appear to be associated with a limited prognosis. So far, no data is available on whether elevated light chains are also important in patients with PID or PID/AATD who often suffer from chronic inflammation. Future investigations might answer this question.

To our knowledge, there are only few publications regarding AATD in patients who fulfill PID criteria. One study reported on AAT allele frequencies in a cohort of CVID patients [24]. In addition, there are only four case reports describing AATD coexisting with hypogammaglobulinemia [19–23]. Against this background, our case series is the largest and first to describe AATD in a cohort of PID patients including two patients with a homozygous AATD coexisting with PID.

Sansom et al. assessed AAT S and Z deficiency allele frequencies in 43 CVID patients [24]. Whereas phenotype MZ was found in three patients, phenotypes MS and SZ were identified in only one patient. With one exception, bronchiectases were detected in all patients with heterozygous AATD and in 21 of 38 patients (55%) without AATD. Patients had not been evaluated for further pulmonary morbidity (i.e., obstruction/restriction and emphysema).

In addition, the authors compared their patient population of 43 CVID patients with a control group of 70 individuals. Regarding the distribution of AAT genes, they found no significant difference between both groups. Subgroup analysis of 26 of 43 CVID patients with bronchiectasis revealed a higher Z allele frequency (0.077 vs. 0.022) compared to healthy controls, yet, there was no relevant difference (*P*=0.15). The authors concluded that, due to the relatively small number of cases, investigations did not provide information on lung damage due to AATD and that studies with a larger number of patients are required to further address this issue [24]. Another study involving 40 patients with primary antibody deficiency was also unable to show a significantly increased frequency for the occurrence of the Z allele in the patient population due to the small number of cases [25].

With regard to our study group, pulmonary morbidity was not found to be more frequent in PID patients with AATD compared to PID patients with normal AAT serum levels. Likewise, there was no difference in the subgroup of patients with CVID. Notably, in contrast to Sansom et al., in our patient population, bronchiectasis was not identified in any of the AAT deficient PID patients but was also present in PID patients without AATD.

In terms of Z allele frequency in PID patients, our data do not allow accurate calculation because AAT genotyping was not performed in the whole cohort but only in patients with low AAT serum levels. However, taking into account the fact that in Germany estimated percentage for genotype PI∗ZZ and PI∗MZ is 0.01% and 1.9% [16], the corresponding prevalence in our patient population of 110 PID patients for PI∗ZZ and PI∗MZ is 1.8% (2 patients) and 4.5% (5 patients), respectively, indicating a possible pathological relationship between AATD and PID. In this context, our literature review supports the hypothesis that an increased coexistence of AATD and PID is conceivable. Of course, coexistence has been described only sporadically, and this hypothesis needs to be proven in a larger PID patient cohort.

### 4.2. Impact of AATD on PID

Generally, up to 35% of patients with homozygous AATD do not develop clinical symptoms [9]. So far, the reasons for the lack of a direct relationship between the genotype and phenotype are not understood. Heterozygous AATD individuals are usually considered to develop clinical symptoms rarely. Yet, even heterozygous AATD is discussed to be associated with pulmonary morbidity (i.e., airway obstruction) in the presence of certain risk factors such as smoking and chronic inflammation [31–33]. Indeed, heterozygous AATD might also worsen clinical symptoms in case of pulmonary inflammation due to recurrent infections in PID patients. With regard to our patient population, there is only one patient with heterozygous AATD and CVID who developed emphysema and chronic obstructive disease despite adequate immunoglobulin substitution. The same applies to patient #2. In contrast, despite rejection of immunoglobulin substitution and recurrent airway infections, patient #1 did not experience further deterioration in lung function. However, this patient is still of younger age and has never smoked before. Without doubt, in the majority of patients, development of pulmonary emphysema and airway obstruction in AATD is strongly associated with smoking due to smoking-related recruitment of neutrophils to the lungs [34]. In turn, AAT protects lung tissue from neutrophil-dependent proteolytic destruction. Therefore, risk for pulmonary morbidity is increased in smoking AATD patients with ZZ or MZ phenotype. However, pulmonary morbidity also develops in nonsmokers because AAT affects tissue inflammation in different ways. Recently, apart from its function as serine protease inhibitor, AAT is reported to modulate local and systemic inflammatory processes by modifying cytokine release [35] and by suppressing neutrophil activation and migration [36]. Moreover, AATD was attributed to generate an adaptive immune inflammation in the lung, similar to what is observed in COPD, comprising CD4+ and CD8+ T-cells, B-lymphocytes, and lymphoid follicles [37]. Therefore, AAT has strongly been considered to function as an anti-inflammatory protein [38]. Interestingly, most recently, AAT substitution was efficaciously performed in severe acute graft versus host disease patients supporting the idea that AAT exerts potent anti-inflammatory effects [39]. Reduced anti-inflammatory ability in AATD patients might therefore contribute to chronic lung damage, especially in the context of PID.

In direct comparison of Sansom's study with our data, it is difficult to conclude that AATD supports development of pulmonary morbidity in PID/CVID patients. Moreover, it cannot be estimated how much PID or AATD contributes to pulmonary morbidity since both diseases lead to similar clinical symptoms and functional limitations. Nevertheless, AATD might explain why pulmonary morbidity occurs in a subgroup of PID patients despite adequate immunoglobulin substitution therapy even in the setting of heterozygous AATD in some cases. Recurrent infections and consequent chronic pulmonary inflammation may therefore have an additive effect on tissue damage in the presence of reduced serum AAT levels with impaired anti-inflammatory capacity. Hence, we suggest testing for AATD in PID patients to identify patients with a potentially increased risk of pulmonary morbidity.

### 4.3. Limitations

One limitation of our analysis is the small number of cases. Consequently, it is difficult to confirm the extent to which AATD contributes to pulmonary morbidity in patients with PID. Hence, larger studies are required.

A second limitation of our study is its retrospective character. Serum AAT levels were only available for 110 patients of our entire group of 149 PID patients. A third limitation is due to the fact that AAT is an acute phase reactant. Since it was usually determined only once in each patient, the frequency of nonidentified AATD patients could even be higher in the presence of systemic inflammation. Additionally, in terms of pulmonary morbidity, future studies should ideally incorporate lung function tests and routine chest imaging of all patients irrespective of clinical symptoms. To rule out AATD, determination of AAT serum levels should be measured at least once, and controls might repeatedly be performed in case of increased inflammatory serum parameters.

Since AATD might not be detected in PID patients, a determination of AAT serum levels should be performed to rule out AATD. Of course, prospective evaluation is mandatory and could support the idea of a general screen for AATD in PID patients. Conversely, PID should also be excluded in patients with proven AATD and recurrent infections. In this context, case #2 suggests that successful treatment of PID might be beneficial for this group of patients. However, to our knowledge, this issue has not been addressed in current AATD guidelines.

In summary, two patients with homozygous and five patients with heterozygous AATD were identified in a group of 110 PID patients. However, it is difficult to demonstrate a possible linkage of both diseases even though AATD might contribute to pulmonary morbidity in PID patients. Whether the coexistence is often underdiagnosed needs further clarification. Consequently, prospective database analysis of larger patient cohorts with concomitant PID and AATD is mandatory to elucidate the relationship and clinical course of both diseases.

## 5. Conclusions

In Germany, the estimated percentage for PI∗ZZ and PI∗MZ is 0.01% and 1.9%, respectively. As demonstrated, the ratio in our study group was even higher. We identified seven patients with AATD. Since AATD contributes to pulmonary morbidity in PID patients, systematic underdiagnosis of the coexistence might yield a strong clinical impact. Hence, AAT analysis should be offered to all patients with confirmed PID diagnoses. To strengthen this finding, we suggest the investigation of larger databases.

## Figures and Tables

**Table 1:** Coexistence of AATD and PID.

Authors	Study patients (*n*)	Age (years)	AAT phenotype	Immune deficiency	Pulmonary morbidity
Gupta et al. [19]	1	89	PI∗ZZ	IgG/IgM hypogammaglobulinemia	Bronchiectasis, emphysema, obstruction, pulmonary embolus
Elsensohn et al. [20]	1	36	PI∗ZZ	IgG/IgM hypogammaglobulinemia	Pulmonary embolus
Casterline et al. [21]	1	54	PI∗ZZ	Selective IgA deficiency	Bronchiectasis, emphysema
Phung et al. [22]	1	34	PI∗ZZ	CVID	Obstruction, emphysema
Phung et al. [23]	3	NA	PI∗MZ	Hypogammaglobulinemia	NA
	5	NA	PI∗MS	Hypogammaglobulinemia	NA
	1	NA	PI∗SZ	Hypogammaglobulinemia	NA
Sansom et al. [24]	1	71	PI∗SZ	CVID	Bronchiectasis
	1	5	PI∗MZ	CVID	Bronchiectasis
	1	22	PI∗MZ	CVID	Bronchiectasis
	1	32	PI∗MZ	CVID	Bronchiectasis
	1	33	PI∗MS	CVID	
Fazlollahi et al. [25]	1	NA	PI∗SS	CVID	
	1	NA	PI∗MZ	CVID	
	1	NA	PI∗MZ	CVID	Bronchiectasis
Evers et al.	1	25	PI∗ZZ	CVID	
	1	45	PI∗ZZ	Unclassified antibody deficiency	Obstruction, emphysema
	1	37	PI∗MZ	CVID	Obstruction, emphysema
	1	47	PI∗MZ	Selective IgM deficiency	
	1	54	PI∗MZ	Selective IgM deficiency	Obstruction
	1	18	PI∗MZ	Isolated IgG subclass deficiency	Panbronchiolitis
	1	50	PI∗MZ	Unclassified antibody deficiency	

**Table 2:** Baseline characteristics of 110 patients with measured AAT levels.

	PID patients without AATD (*n* = 103)	PID patients with AATD (*n* = 7)	
		ZZ (*n* = 2)	MZ (*n* = 5)
Clinical parameters			
Median age (± SD)	49 (±16)	39 (±10)	43 (±13)
Male gender, *N* (%)	40 (39)	2 (100)	2 (40)
Female gender, *N* (%)	63 (61)	0	3 (60)

Alpha 1-antitrypsin level (mg/dl)			
Median	147.5	39	86
Range	97−313	19−59	74−90

Respiratory parameters			
Obstruction, *N* (%)	28 (27)	2 (100)	2 (40)
Restriction, *N* (%)	9 (9)	0	0

Radiologic parameters			
Bronchiectasis, *N* (%)	18 (17)	0	0
Emphysema, *N* (%)		1 (50)	1 (20)

PID			
CVID	41 (40)	1 (50)	1 (20)
IgA with IgG subclass deficiency	5 (5)	0	0
Unclassified antibody deficiency	30 (29)	1 (50)	1 (20)
Isolated IgG subclass deficiency	16 (16)	0	1 (20)
Hyper-IgM Syndrome	1 (1)	0	0
Hyper-IgE Syndrome	1 (1)	0	0
Selective IgM deficiency	6 (6)	0	2 (40)
Selective IgA deficiency	3 (3)	0	0

## Data Availability

The retrospective data used to support the findings of this study are included within the article.
